# Differences in Left–Right Vestibulo-Ocular Reflex Gain in the Video Head Impulse Test

**DOI:** 10.3390/jcm15176882

**Published:** 2026-09-05

**Authors:** Jihye Rhee, Ja-Won Koo

**Affiliations:** 1 Department of Otorhinolaryngology-Head & Neck Surgery, Veterans Health Service Medical Center, Gangdong-gu, Seoul 05368, Republic of Korea; jrheeent@gmail.com; 2 Department of Otorhinolaryngology-Head & Neck Surgery, Seoul National University Bundang Hospital, Seoul National University College of Medicine, Bundang-gu, Seongnam-si 13620, Gyeonggi-do, Republic of Korea

**Keywords:** video head impulse test, vestibulo-ocular reflex gain, slow-phase velocity, left–right difference

## Abstract

**Objectives:** The objective of this study was to evaluate left–right symmetry of vestibulo-ocular reflex (VOR) gain on the video head impulse test (vHIT) and its diagnostic implications relative to caloric test results. **Methods**: Patients presenting with dizziness who underwent a bithermal caloric test and vHIT were retrospectively analyzed. Of 764 patients identified, nine with bilateral vestibular hypofunction were excluded, leaving 755 patients for analysis (451 showing symmetric caloric responses; 304 with unilateral vestibular hypofunction (UVH)). Horizontal VOR gain was compared between directions in subjects with normal caloric responses and according to the affected side in patients with UVH. Subgroup analyses were performed according to caloric weakness severity. Caloric findings were analyzed according to horizontal VOR gain patterns, and the diagnostic performance of isolated unilateral low VOR gain (<0.8) for ipsilateral UVH was evaluated. **Results:** In subjects with normal caloric results, rightward VOR gain was significantly higher than leftward (1.03 [0.95–1.11] vs. 0.93 [0.87–0.99]; *p* < 0.001). In patients with UVH, ipsilateral VOR gain was significantly greater in right-sided than in left-sided UVH at comparable levels of caloric weakness. Subgroup analysis showed that this difference was significant in patients with mild-to-moderate caloric weakness. Isolated right low VOR gain was associated with right UVH in 90.5% of cases, whereas isolated left low VOR gain was associated with left UVH in 54.5%; the positive predictive value (PPV) differed significantly between sides (90.5% vs. 54.5%; *p* = 0.004). **Conclusions:** Horizontal VOR gain on vHIT showed a left–right asymmetry associated with side-dependent diagnostic performance. When the same VOR gain cutoff was applied to both sides, isolated right low VOR gain had a substantially higher PPV for ipsilateral UVH than isolated left low VOR gain. These findings indicate that the direction of head impulse should be considered when interpreting VOR gain.

## 1. Introduction

The primary vestibular afferents exhibit spontaneous resting activity, allowing bidirectional modulation through excitation and inhibition. During head rotation, excitation of the ipsilateral vestibular afferents is accompanied by inhibition of the contralateral side, thereby generating the vestibulo-ocular reflex (VOR). In unilateral vestibular disorders such as vestibular neuritis or the irritative stage of Ménière’s disease, disruption of this physiological balance produces asymmetric vestibular input, resulting in vertigo. Accordingly, symmetric vestibular function is a fundamental principle of the normal vestibular system and a prerequisite for the interpretation of vestibular function tests.

The principle of left–right symmetry is reflected in the major vestibular function tests currently used in clinical practice. In the bithermal caloric test, each lateral semicircular canal is stimulated independently using warm and cool irrigation, and vestibular asymmetry is quantified as canal paresis (CP) and directional preponderance according to the Jongkees formula [1]. Similarly, rotational chair testing evaluates vestibular responses during clockwise and counterclockwise rotations, with left–right asymmetry expressed as directional preponderance [1]. The video head impulse test (vHIT), which quantitatively evaluates semicircular canal function by measuring the VOR gain during rapid passive head impulses, also assumes that equivalent head impulses delivered to both directions produce symmetric VOR gains under normal conditions.

However, accumulating evidence suggests that VOR gain measured by vHIT may not be inherently symmetric. Several studies have reported systematic left–right differences in horizontal VOR gain, even in healthy subjects without vestibular disorders [2,3,4]. Various mechanisms have been proposed to explain this asymmetry, including differences between outward and inward head impulses, extraocular muscle mechanics, examiner handedness, and the use of monocular recording systems [3,4,5]. Although these studies demonstrated left–right asymmetry in VOR gain, they primarily focused on the technical characteristics of the vHIT system or normative data, with relatively little attention paid to its clinical implications.

To clarify this issue, we evaluated the left–right symmetry of horizontal VOR gain measured by vHIT and investigated its diagnostic implications.

## 2. Materials and Methods

### 2.1. Study Design and Population

This was a retrospective study of patients (20–65 years old) who presented to one of two tertiary hospitals located in Seoul, Republic of Korea, and Seongnam, Gyeonggi Province, Republic of Korea, with a chief complaint of dizziness between January 2020 and March 2021. They underwent vHIT followed by a bithermal caloric test on the same day. Patients with bilateral vestibular hypofunction or who failed to complete the caloric test were excluded. As this study was a retrospective analysis of medical records, informed consent was waived. The study protocol was approved by the institutional review board of Veterans Health Service Medical Center (approval number 2022-09-002-003).

### 2.2. Bithermal Caloric Test

The test was performed in the supine position with the head elevated 30 degrees using a NCI480 water caloric stimulator (ICS Medical, Schaumburg, IL, USA). The order of irrigation was as follows: right ear with cool water, left ear with cool water, right ear with warm water, and left ear with warm water. Each irrigation lasted for 30 s with a flow rate of 300 mL/min for cold (30 °C) and warm (44 °C) water. Eye movements were recorded using a videonystagmography system (ICS Chart 200 with VG-40 Video Goggles; GN Otometrics, Taastrup, Denmark) equipped with two infrared cameras, one positioned above each eye. The maximum slow-phase velocity (SPV) of induced nystagmus for 10 s was averaged after each irrigation, and CP was determined according to Jongkees’ formula. Unilateral vestibular hypofunction (UVH) was diagnosed when the CP score was more than 25%. Bilateral vestibular hypofunction was diagnosed when the sum of the maximum SPV induced by the four caloric irrigations was less than 12°/s. For subgroup analysis, the UVH patients were divided into three subgroups based on caloric weakness: weakness ≤ 40% (subgroup 1), >40% to 70% (subgroup 2), and severe vestibular hypofunction with caloric weakness > 70% (subgroup 3).

### 2.3. Video Head Impulse Test

Before the test, participants were asked to stare at a fixed target 1 m away. The examiners lightly grasped the participant’s head and applied rapid head rotations to assess the function of the three semicircular canals. Rightward and leftward impulses were delivered in random order. Proper head rotation was determined by whether the peak acceleration was between 750 and 6000°/s^2^. Each head rotation was repeated at least 10 times per direction. Head rotation and associated eye movements were analyzed using a vHIT system (ICS Impulse^®^; GN Otometrics). The movement of the eyeball was recorded using a monocular camera mounted on the right side of the participant’s face. The ratio of the area under the velocity curves of the head and eye was calculated as the VOR gain for each side and direction.

### 2.4. Analyzed Parameters and Statistics

The normality of continuous variables was assessed using the Shapiro–Wilk test. Appropriate parametric or nonparametric tests were selected based on data distribution and the structure of each analysis. Demographic data were compared between the normal and UVH groups using the chi-square test for sex differences and the Mann–Whitney U test for age. CP values were compared between the right and left UVH groups using the Mann–Whitney U test. In the normal, right UVH, and left UVH groups, two parameters were analyzed separately: the sum of the SPVs of the warm and cold stimulations and the horizontal VOR gain. For each parameter, the values of the right and left sides were compared using the Wilcoxon signed-rank test. For these paired comparisons, effect sizes (r) were calculated, and 95% confidence intervals for the median paired right-left differences were estimated using bootstrap resampling. To assess potential effects of age and sex on left–right VOR gain asymmetry, additional linear regression analyses were performed within the normal, right UVH, and left UVH groups, with the left–right VOR gain difference as the dependent variable and age and sex as independent variables. In the previously defined subgroups 1, 2, and 3, caloric weakness, VOR gain, and the sum of the SPVs on the affected side were separately compared between the right and left UVH groups using the Mann–Whitney U test. In patients with UVH, an additional multivariable linear regression analysis was performed with ipsilateral VOR gain as the dependent variable and UVH side, age, sex, and caloric weakness as independent variables. Patients were additionally classified according to horizontal VOR gain patterns on vHIT as bilaterally normal, isolated right low VOR gain, isolated left low VOR gain, or bilateral low VOR gain, using 0.8 as the cutoff for low VOR gain. Sensitivity, specificity, positive predictive value (PPV), and negative predictive value (NPV) of isolated unilateral low VOR gain for ipsilateral UVH were calculated. The PPVs of isolated right and left low VOR gain for ipsilateral UVH were compared using Fisher’s exact test. All statistical analyses were performed using IBM SPSS Statistics version 21.0 (IBM Corp., Armonk, NY, USA). Statistical significance was defined as a *p*-value < 0.05. To account for multiple comparisons, Bonferroni correction was applied within each analysis family.

## 3. Results

### 3.1. Demographic Data

In all, 764 patients completed both the caloric test and vHIT. Excluding 9 patients with bilateral vestibular hypofunction, 451 patients with normal vestibular function and 304 patients with UVH, as determined by the caloric test, were included. The demographic characteristics of the study population, including age and sex distribution, are presented in Table 1. There were 126 and 178 patients in the right UVH and left UVH groups, respectively. The median CP values of the right UVH group and left UVH group were 50.3 (33.3–70.1) and 46.5 (33.3–69.3), respectively (*p* = 0.371).

### 3.2. Left–Right Differences in the SPV and VOR Gain

**Figure 1:**
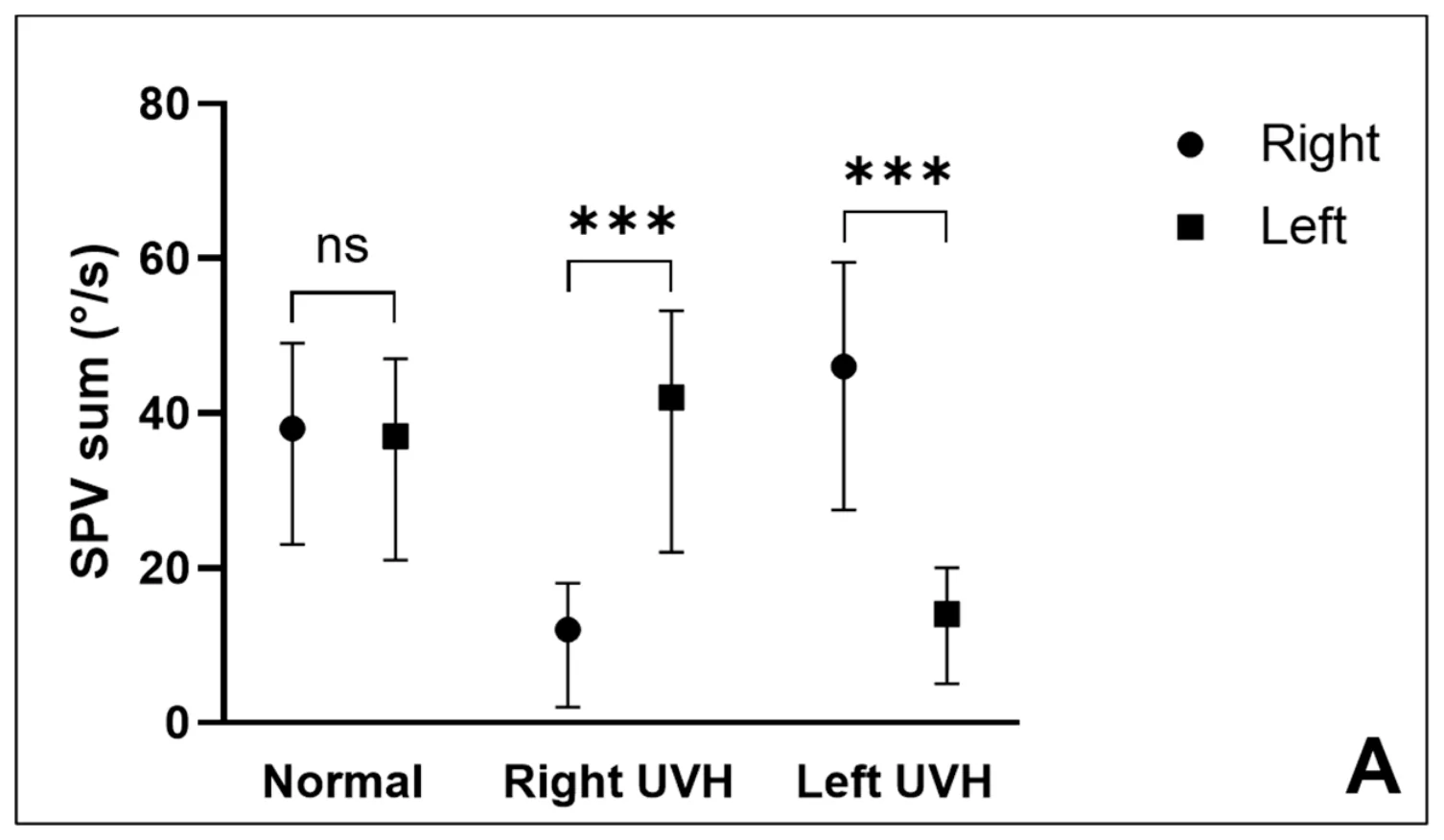

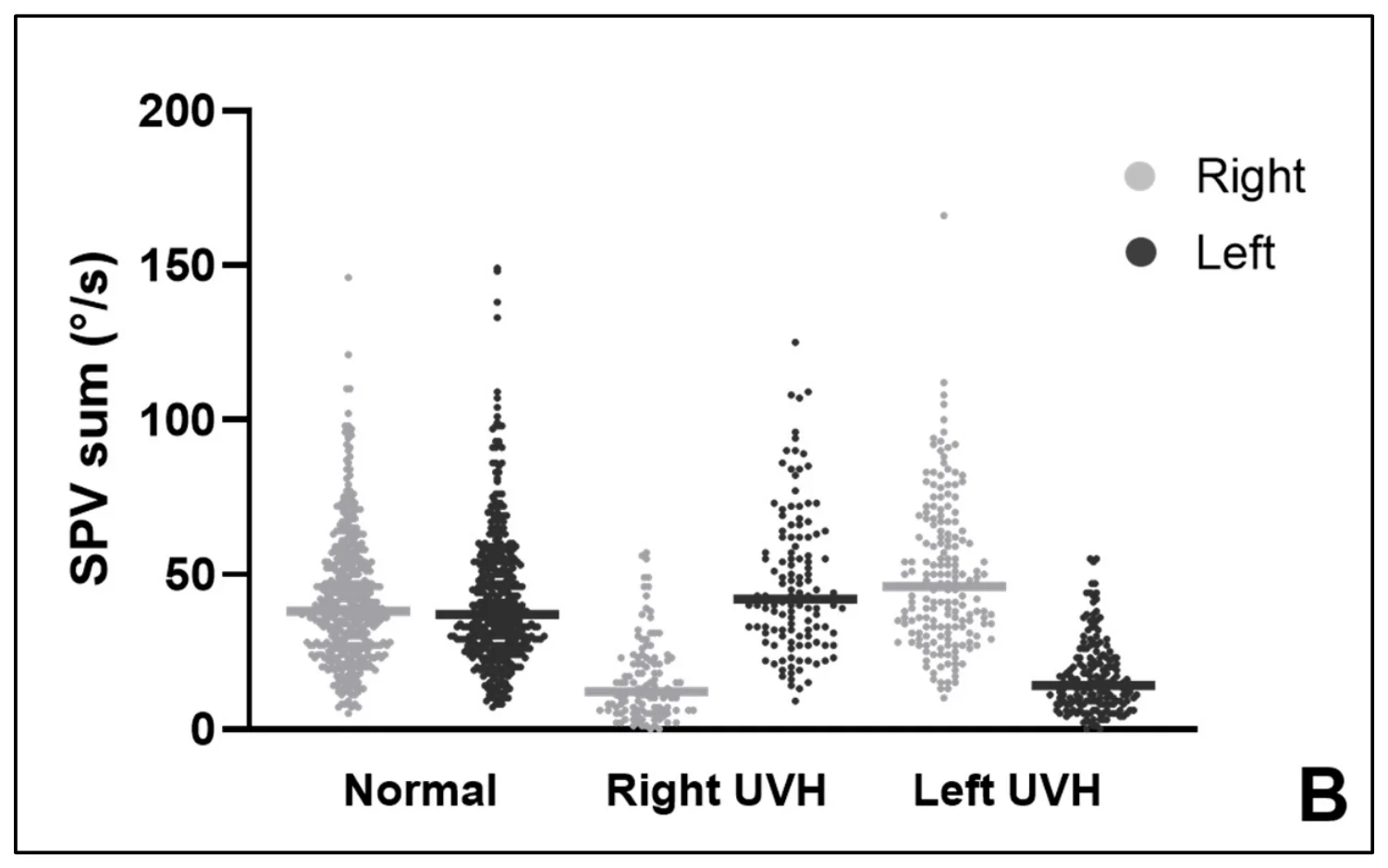
Comparison of the sum (cold and warm irrigations) of slow-phase velocities (SPVs) between the right and left ears. (**A**) Median SPV sums in the normal, right unilateral vestibular hypofunction (UVH), and left UVH groups. Symbols represent median values, and error bars indicate the interquartile range (Q1–Q3). (**B**) Individual SPV sum values in the same groups. Horizontal lines indicate median values. SPV sums (°/s) were compared between the right and left ears within each group using the Wilcoxon signed-rank test. *** *p* < 0.001; ns, not significant.

The SPV sums of cold and warm stimulation were not significantly different between the right and left ears in the normal group (38.0 [27.0–53.0] vs. 37.0 [27.0–53.0], respectively; *p* = 0.596). In the right and left UVH groups, the SPV sum on the affected side was significantly lower than that on the unaffected side (12.0 [6.0–22.0] vs. 42.0 [30.8–62.0] and 14.0 [8.0–23.0] vs. 46.0 [32.5–64.5], respectively; *p* < 0.001 for both) (Figure 1A,B). The median paired right-left differences were 0.0 (95% CI, −1.0 to 1.0), −28.0 (95% CI, −31.5 to −25.5), and 28.0 (95% CI, 25.5 to 31.5) in the normal, right UVH, and left UVH groups, respectively, with corresponding effect sizes of r = 0.03, 0.87, and 0.87.

**Figure 2:**
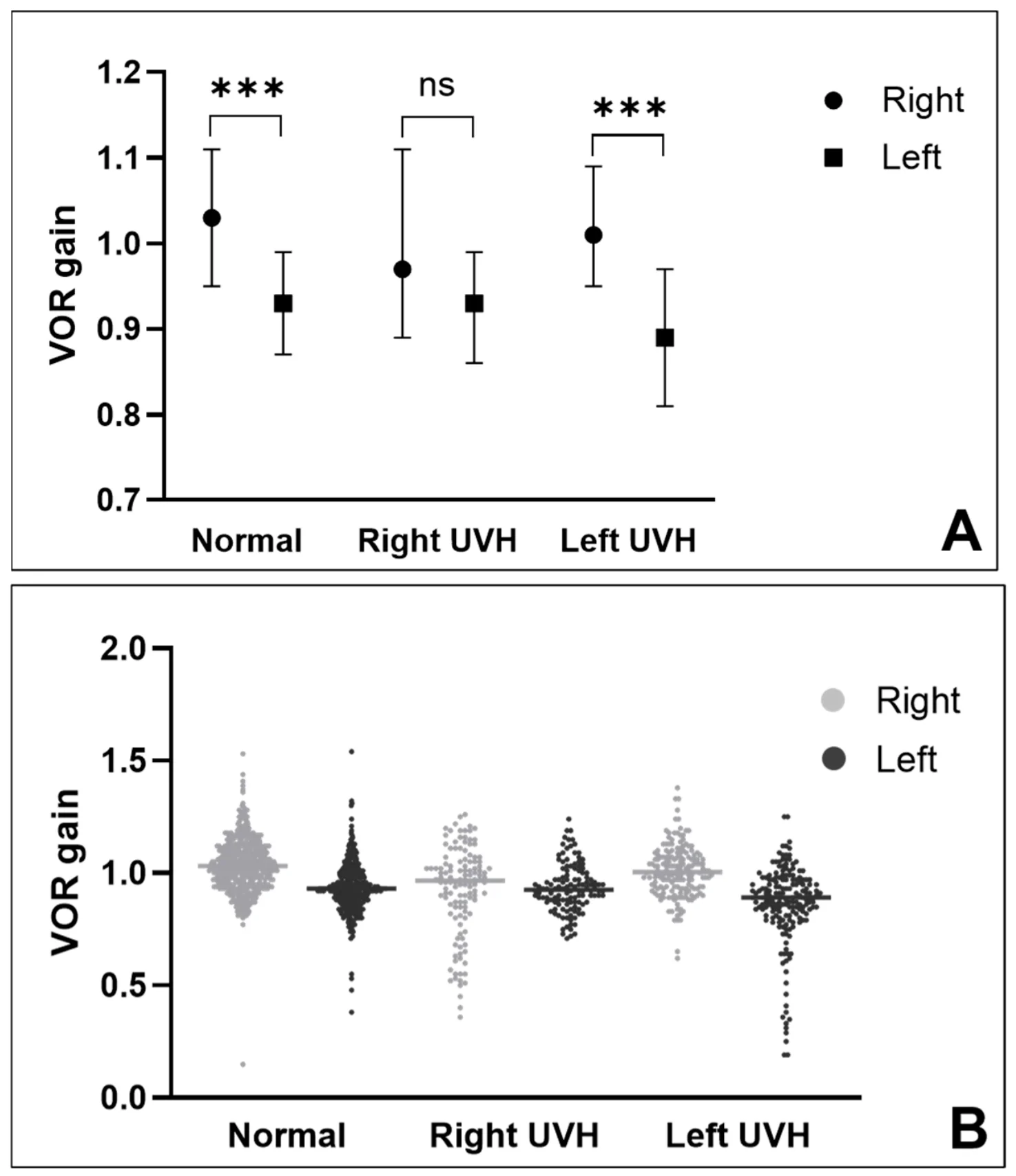
Comparison of horizontal vestibulo-ocular reflex (VOR) gain between the right and left sides. (**A**) Median horizontal VOR gains in the normal, right unilateral vestibular hypofunction (UVH), and left UVH groups. Symbols represent median values, and error bars indicate the interquartile range (Q1–Q3). (**B**) Individual horizontal VOR gain values in the same groups. Horizontal lines indicate median values. Horizontal VOR gains were compared between the right and left sides within each group using the Wilcoxon signed-rank test. *** *p* < 0.001; ns, not significant.

However, the VOR gain on each side exhibited unexpected patterns. In the normal group, the right VOR gain was significantly higher than the left VOR gain (1.03 [0.95–1.11] vs. 0.93 [0.87–0.99], respectively; *p* < 0.001). In the right UVH group, there was no significant difference in the VOR gain between the affected and unaffected sides (0.97 [0.83–1.05] vs. 0.93 [0.87–1.00], respectively; *p* = 0.316). In the left UVH group, the left VOR gain was significantly lower than the right VOR gain (0.89 [0.81–0.97] vs. 1.01 [0.93–1.07], respectively; *p* < 0.001) (Figure 2A,B). The median paired right-left differences were 0.10 (95% CI, 0.09 to 0.10), 0.02 (95% CI, 0.005 to 0.05), and 0.11 (95% CI, 0.095 to 0.13) in the normal, right UVH, and left UVH groups, respectively, with corresponding effect sizes of r = 0.82, 0.09, and 0.83. In additional linear regression analyses, the observed left–right VOR gain differences were not significantly associated with age or sex in any of the three groups (all *p* > 0.05).

### 3.3. Comparison of Affected-Side SPV Sum and VOR Gain Between Right and Left UVH According to the Severity of Caloric Weakness

To examine how the difference between right and left VOR gains varied depending on the affected side and severity of caloric weakness, subgroup analysis was performed according to the severity of caloric weakness. The caloric weakness was not significantly different between the right UVH and left UVH in each subgroup (Table 2).

**Figure 3:**
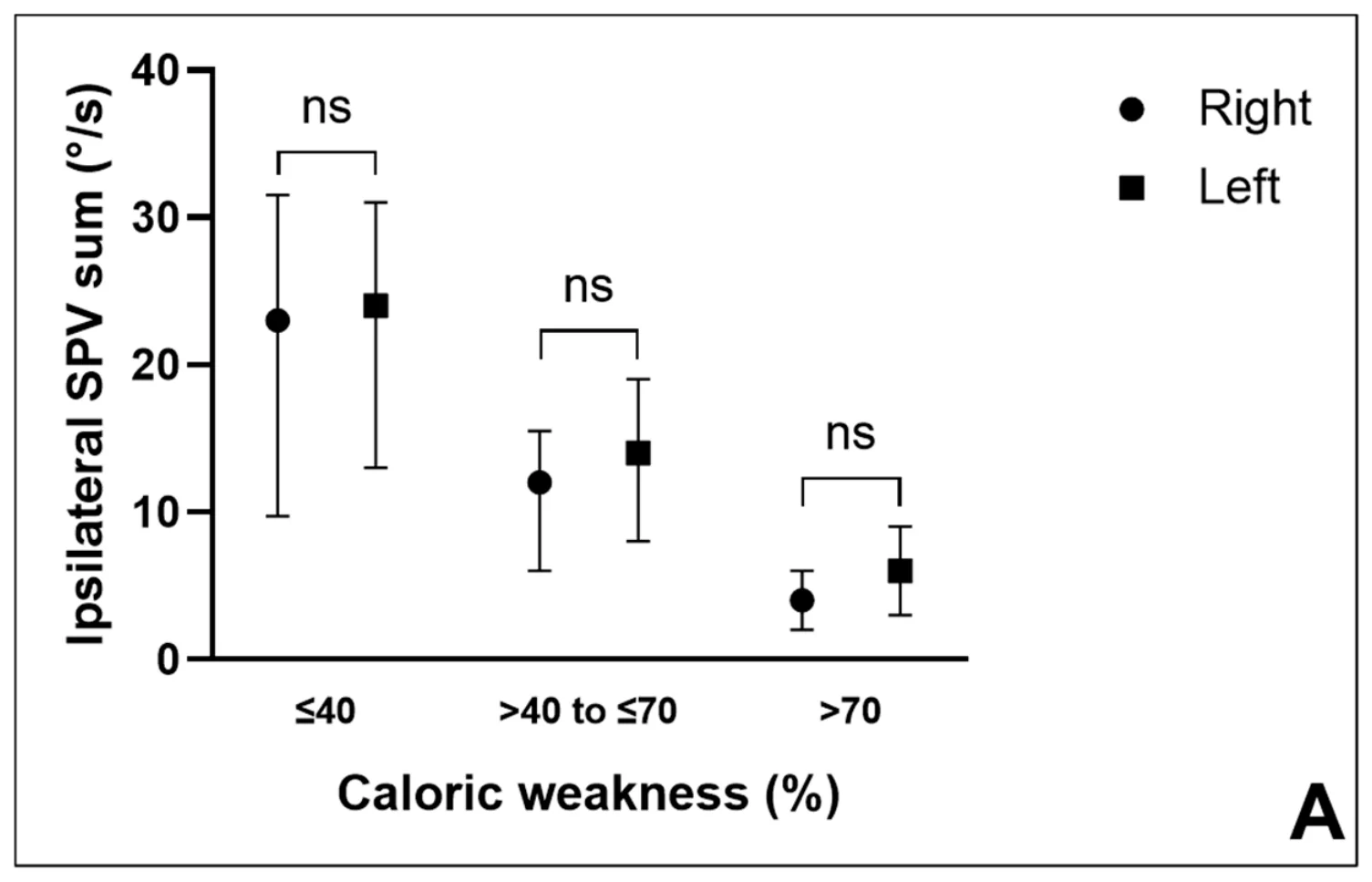

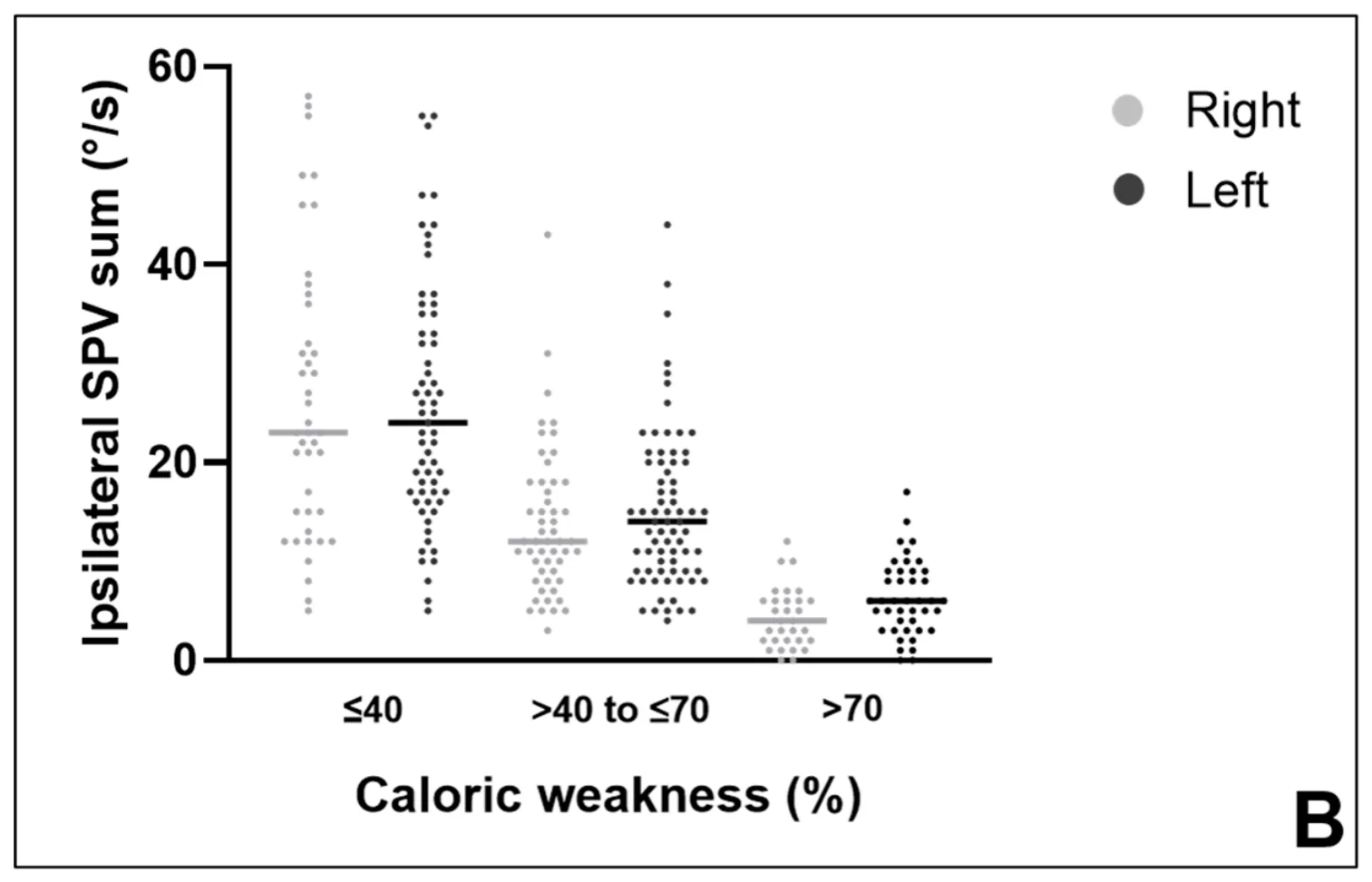
Ipsilateral slow-phase velocity (SPV) sums according to the severity of caloric weakness. (**A**) Median ipsilateral SPV sums in the right unilateral vestibular hypofunction (UVH) and left UVH groups across subgroups 1, 2, and 3. Symbols represent median values, and error bars indicate the interquartile range (Q1–Q3). (**B**) Individual ipsilateral SPV sum values in the same subgroups. Horizontal lines indicate median values. Ipsilateral SPV sums (°/s) were compared between the right and left UVH groups within each subgroup using the Mann–Whitney U test. Bonferroni correction was applied for multiple comparisons. ns, not significant.

The sum of the SPVs on the affected side did not differ between the right UVH and left UVH groups in subgroups 1 and 2 (23.0 [14.5–36.3] vs. 24.0 [17.0–35.0], respectively, in subgroup 1, *p* = 0.992; 12.0 [8.5–18.0] vs. 14.0 [9.0–20.0], respectively, in subgroup 2, *p* = 0.361). Although a difference was observed in subgroup 3 before correction (4.0 [2.0–6.0] vs. 6.0 [3.0–9.0], respectively; *p* = 0.021), it was no longer significant after Bonferroni correction (Figure 3A,B).

**Figure 4:**
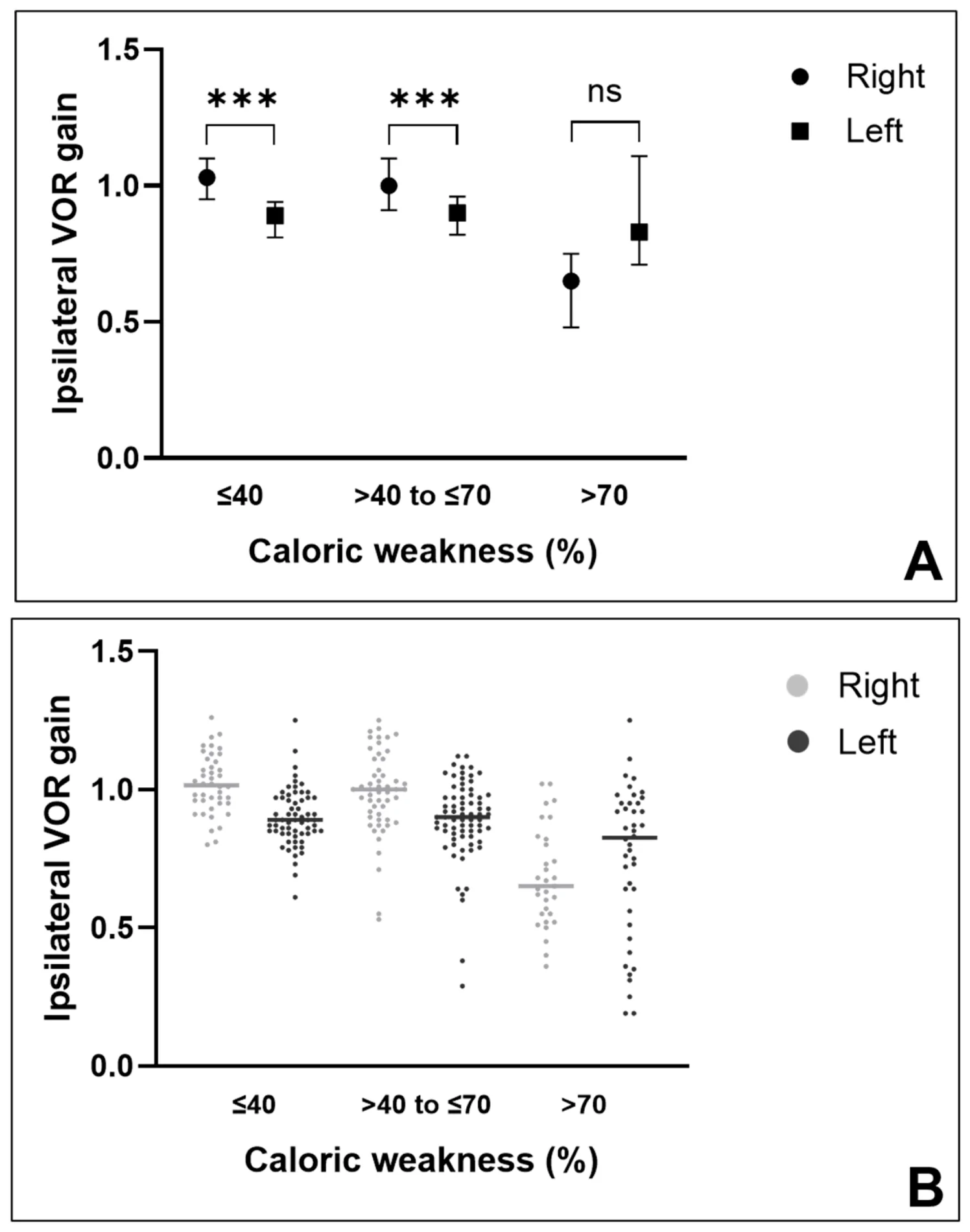
Ipsilateral horizontal vestibulo-ocular reflex (VOR) gain according to the severity of caloric weakness. (**A**) Median ipsilateral horizontal VOR gains in the right unilateral vestibular hypofunction (UVH) and left UVH groups across subgroups 1, 2, and 3. Symbols represent median values, and error bars indicate the interquartile range (Q1–Q3). (**B**) Individual ipsilateral horizontal VOR gain values in the same subgroups. Horizontal lines indicate median values. Ipsilateral horizontal VOR gains were compared between the right and left UVH groups within each subgroup using the Mann–Whitney U test. Bonferroni correction was applied for multiple comparisons. *** *p* < 0.001; ns, not significant.

In contrast to the SPV findings, the VOR gain showed a different pattern. The VOR gain on the affected side in subgroups 1 and 2 was significantly higher in the right UVH group than in the left UVH group (1.02 [0.95–1.10] vs. 0.89 [0.84–0.97], respectively, in subgroup 1; 1.00 [0.90–1.09] vs. 0.90 [0.84–0.98], respectively, in subgroup 2; *p* < 0.001 for both), and these differences remained significant after Bonferroni correction. However, there was no significant difference in the affected-side VOR gain between the right and left UVH groups in subgroup 3 (0.65 [0.55–0.82] vs. 0.83 [0.55–0.95], respectively; *p* = 0.095) (Figure 4A,B). In an additional multivariable linear regression analysis, UVH side remained significantly associated with ipsilateral VOR gain after adjustment for age, sex, and caloric weakness (B = −0.066; 95% CI, −0.102 to −0.029; *p* = 0.001).

### 3.4. Caloric Findings According to Horizontal VOR Gain Patterns on vHIT

In the preceding analyses, VOR gain on vHIT showed different patterns according to the side of UVH as determined by the caloric test. As an additional analysis, we examined caloric findings according to horizontal VOR gain patterns on vHIT, using a VOR gain <0.8 as the criterion for low gain. Among 21 patients with isolated right low VOR gain, 19 (90.5%) had right UVH, 1 (4.8%) had normal caloric findings, and 1 (4.8%) had left UVH. In contrast, among 66 patients with isolated left low VOR gain, 36 (54.5%) had left UVH, while 27 (40.9%) had normal caloric findings and 3 (4.5%) had right UVH. Among 12 patients with bilateral low VOR gain, 7 (58.3%) had right UVH, 4 (33.3%) had left UVH, and 1 (8.3%) had normal caloric findings (Table 3).

The diagnostic performance of isolated unilateral low VOR gain for ipsilateral UVH is shown in Table 4. Isolated right low VOR gain showed a sensitivity of 15.1%, specificity of 99.7%, PPV of 90.5%, and NPV of 85.4% for right UVH. Isolated left low VOR gain showed a sensitivity of 20.2%, specificity of 94.8%, PPV of 54.5%, and NPV of 79.4% for left UVH. The PPV for ipsilateral UVH was significantly higher for isolated right low VOR gain than for isolated left low VOR gain (90.5% vs. 54.5%, respectively; *p* = 0.004, Fisher’s exact test).

## 4. Discussion

Some previous studies have reported left–right asymmetry in VOR gain measured by vHIT, whereas the clinical implications of this asymmetry in patients with vestibular hypofunction have not been fully addressed. To clarify its clinical significance, we retrospectively analyzed the relationship between VOR gain and caloric weakness in 755 patients, including 451 with normal caloric responses, 126 with right UVH, and 178 with left UVH. The right VOR gain was significantly larger than the left VOR gain in the normal group. In contrast, despite the definite left–right asymmetry demonstrated by the caloric test in the right UVH group, there was no significant decrease in the right VOR gain compared with the left VOR gain. Analysis of the ipsilateral VOR gain in the UVH group showed consistent results. The right VOR gain in the right UVH was significantly larger than the left VOR gain in the left UVH. Subgroup analysis showed that this difference was mainly observed in patients with mild-to-moderate UVH (subgroups 1 and 2).

The preceding analyses demonstrated that VOR gain patterns differed according to the side of UVH as defined by the caloric test. To further examine the diagnostic implications of vHIT when interpreted as an initial vestibular function test, we analyzed the distribution of caloric findings according to horizontal VOR gain patterns on vHIT. This analysis may reflect a clinically relevant scenario in which a clinician evaluates a patient with dizziness after an abnormal VOR gain has already been identified on vHIT. Using a horizontal VOR gain <0.8 as the criterion for low gain, isolated right low VOR gain was associated with right UVH in 90.5% of cases, whereas isolated left low VOR gain was associated with left UVH in only 54.5% of cases. The PPV for ipsilateral UVH was significantly higher for isolated right low VOR gain than for isolated left low VOR gain. Despite the marked difference in PPV, isolated unilateral low VOR gain showed low sensitivity for ipsilateral UVH on both sides. Therefore, VOR gain on vHIT should not be interpreted in isolation, and its side-dependent characteristics should be considered together with other vestibular test findings.

Several mechanisms have been proposed to explain the left–right asymmetry of VOR gain measured by vHIT. Previous studies suggested that examiner handedness may influence head impulse velocity and trajectory, potentially introducing systematic differences between rightward and leftward impulses. In the present study, both examiners were right-handed. However, even if examiner handedness resulted in differences in absolute head impulse velocity between directions, VOR gain represents the compensatory eye movement relative to the corresponding head movement rather than the absolute head velocity itself. Therefore, such differences in impulse velocity would not necessarily translate directly into differences in VOR gain. Moreover, a previous study performed by an experienced right-handed examiner reported higher rightward VOR gain despite no significant difference in head velocity between rightward and leftward impulses, suggesting that examiner-related differences in head impulse velocity alone may not fully account for the observed left–right gain asymmetry [6].

Another proposed explanation is the difference between outward and inward head impulses. Outward impulses are initiated from the midline and directed toward the tested side, whereas inward impulses are initiated from a laterally rotated head position and directed toward the midline. The slightly higher VOR gain reported with outward impulses has been attributed to differences in final eye position and the resulting contribution of eye-position signals to the VOR, with a possible additional contribution from the cervico-ocular reflex [3]. To avoid variability associated with different impulse types, all vHIT examinations in the present study were performed using outward head impulses.

Some studies have suggested a possible role of monocular recording in left–right VOR gain asymmetry, with higher gains reported for head impulses toward the side of the recorded eye [4,5]. In a study comparing different vHIT systems, left–right gain asymmetry was observed with monocular recording systems but not with a binocular recording system [4]. In addition, Strupp et al. reported that the direction of the gain asymmetry reversed when the recorded eye was changed, further suggesting an association between recording configuration and directional VOR gain differences [5]. Several mechanisms have been proposed for this difference, including distinct neural pathways involved in adduction and abduction and differences in the mechanical properties of the medial and lateral rectus muscles [3,4,6]. The vHIT system used in the present study also employed monocular recording of right eye movements, and the observed left–right asymmetry may therefore be associated with monocular recording.

While some studies have emphasized the potential directional bias associated with monocular recording [3,5], others have suggested that such differences may have limited effects on clinical classification [4]. In the present study, even in the normal group, the median paired left–right VOR gain difference was 0.10 and was statistically significant. Such a systematic directional difference in individuals with normal caloric responses may be mistaken for pathological asymmetry and should therefore be considered when interpreting vHIT findings. Furthermore, in the right UVH group, despite a median caloric weakness of 50.3%, the median paired left–right VOR gain difference was only 0.02, suggesting that the directional bias in VOR gain could influence the interpretation of right UVH. At the individual-patient level, such an apparently symmetric vHIT pattern in right UVH may give clinicians a falsely reassuring impression and potentially lead to a false-negative interpretation of the vHIT result. This side-dependent difference was notable for cases with mild-to-moderate vestibular hypofunction (subgroups 1 and 2). No significant difference was observed in subgroup 3. The absence of a significant difference may be related to the greater severity of vestibular dysfunction in this subgroup, in which the effect of severe vestibular loss may outweigh the relatively modest directional bias in VOR gain. In addition, although the difference in caloric weakness was not statistically significant, patients with right UVH in subgroup 3 showed numerically greater caloric weakness and lower ipsilateral SPV sums than those with left UVH. This residual difference in vestibular loss severity may have further attenuated the left–right difference in ipsilateral VOR gain. However, because these between-group differences in caloric weakness and SPV were not statistically significant, this interpretation remains speculative.

The optimal cutoff value for horizontal VOR gain on vHIT has not been consistently established across studies. Park et al. proposed a cutoff of 0.875, which provided a sensitivity of 85.7% and a specificity of 79.2% [7]. However, a cutoff of 0.8 has been commonly used in previous studies [2,6,8], and we adopted this conventional threshold in the present study.

Previous studies have investigated the relationship between caloric weakness and reduced VOR gain on vHIT, with several emphasizing the relatively limited sensitivity of vHIT compared with caloric testing. McCaslin et al. reported that a caloric asymmetry of 39.5% was required to optimize discrimination between normal and abnormal vHIT results, suggesting that vHIT abnormalities are more likely to be detected with greater caloric weakness [9]. Bell et al. likewise found abnormal vHIT gain in only 4 of 14 patients with significant CP, and Mezzalira et al. reported substantially more abnormal caloric than vHIT results in patients with chronic vestibular complaints, concluding that caloric testing was more sensitive for detecting vestibular dysfunction [10,11]. In the present study, isolated unilateral low VOR gain also showed low sensitivity for ipsilateral UVH, with sensitivities of 15.1% for right UVH and 20.2% for left UVH, broadly consistent with these previous observations.

In contrast, van Esch et al. reported a relatively high PPV of 88% for abnormal vHIT, together with a sensitivity of 31%, specificity of 98%, and NPV of 73%, indicating that an abnormal vHIT may nevertheless be strongly associated with an abnormal caloric result [8]. Based on our finding that VOR gain patterns differed between right and left UVH, we further evaluated the diagnostic performance of isolated unilateral low VOR gain separately according to side. Among patients with isolated right low VOR gain, 90.5% (19/21) had right UVH, whereas only 54.5% (36/66) of those with isolated left low VOR gain had left UVH, resulting in a marked difference in PPV. Thus, isolated right low VOR gain strongly suggested right UVH, whereas isolated left low VOR gain should be interpreted with greater caution when inferring left UVH. This side-dependent difference indicates that left–right VOR gain asymmetry can have clinically relevant diagnostic implications.

This study had some limitations. First, all vHIT data were obtained using a single monocular system from one manufacturer, which may limit the generalizability of our findings. The left–right asymmetry observed in this study may reflect characteristics of the specific monocular recording system used rather than a universal feature of vHIT. Although monocular vHIT systems are frequently used in both clinical practice and research, our findings should not be assumed to apply uniformly to other vHIT devices or recording configurations. Second, we only analyzed the horizontal plane (i.e., not the vertical plane). The left–right difference was quantified through comparison with caloric test results, which have been proven to be clinically significant over a long period. Therefore, similar to the caloric test, the VOR gain in the horizontal plane was considered the primary analysis target to evaluate the function of the lateral semicircular canal. The present study demonstrated a distinct left–right asymmetry in horizontal VOR gain. Future studies examining potential factors influencing VOR gain in the vertical planes may provide further insight into the characteristics of vHIT systems. Third, the retrospective design limited our ability to evaluate the relationship between the observed vHIT findings and patient-centered clinical outcomes. The present study demonstrated that left–right VOR gain asymmetry was associated with marked side-dependent differences in the PPV of isolated unilateral low VOR gain, providing further information on the diagnostic characteristics of vHIT. However, clinical features such as dizziness handicap, fall risk, and recovery were not systematically assessed or analyzed. Therefore, the implications of the observed asymmetry for patient-centered outcomes remain to be determined in future prospective studies.

## 5. Conclusions

In conclusion, horizontal VOR gain measured by the vHIT system used in this study showed a left–right asymmetry associated with side-dependent diagnostic performance. When the same VOR gain cutoff was applied to both sides, isolated right low VOR gain had a substantially higher PPV for ipsilateral UVH than isolated left low VOR gain. These findings indicate that the direction of head impulse should be considered when interpreting VOR gain.

## Figures and Tables

**Table 1:** Patients’ demographic data.

	Total (*n* = 755)	Normal (*n* = 451)	UVH (*n* = 304)	*p*
Age, years, median (Q1–Q3)	52 (42–59)	50 (40–58)	54 (45–60)	0.001
Male: Female	263:492	153:298	110:194	0.523

UVH, unilateral vestibular hypofunction.

**Table 2:** The severity of caloric weakness among subgroups in right UVH and left UVH. Caloric weakness ≤ 40% was defined as subgroup 1; caloric weakness between >40% to 70% was defined as subgroup 2; and severe vestibular hypofunction with caloric weakness > 70% was defined as subgroup 3.

Caloric Weakness, % (*n*)	Right UVH		Left UVH		*p*
	*n*	Caloric Weakness (%) Median (Q1–Q3)	*n*	Caloric Weakness (%) Median (Q1–Q3)	
≤40 (105)	42	30.9 (27.9–33.3)	63	30.8 (27.3–34.2)	0.937
>40, ≤70 (126)	53	53.8 (45.5–61.9)	73	51.2 (44.8–57.9)	0.164
70< (73)	31	84.6 (75.4–91.1)	42	78.3 (73.7–83.9)	0.081

UVH, unilateral vestibular hypofunction. *p* values were obtained using the Mann–Whitney U test.

**Table 3:** Distribution of caloric findings according to horizontal VOR gain patterns on vHIT. Low VOR gain was defined as a horizontal VOR gain <0.8. Isolated right low VOR gain and isolated left low VOR gain indicate unilateral low VOR gain with a contralateral gain ≥0.8; bilateral low VOR gain indicates VOR gain <0.8 on both sides.

VOR Gain	Normal (*n*)	Right UVH (*n*)	Left UVH (*n*)	Total (*n*)
Normal	422 (64.3%)	97 (14.8%)	137 (20.9%)	656 (100%)
Isolated right low VOR gain	1 (4.8%)	19 (90.5%)	1 (4.8%)	21 (100%)
Isolated left low VOR gain	27 (40.9%)	3 (4.5%)	36 (54.5%)	66 (100%)
Bilateral low VOR gain	1 (8.3%)	7 (58.3%)	4 (33.3%)	12 (100%)

UVH, unilateral vestibular hypofunction. VOR, Vestibulo-ocular reflex.

**Table 4:** Diagnostic performance of isolated unilateral low VOR gain for ipsilateral UVH.

	Isolated Right Low VOR Gain	Isolated Left Low VOR Gain
Sensitivity	15.1%	20.2%
Specificity	99.7%	94.8%
PPV	90.5%	54.5%
NPV	85.4%	79.4%

UVH, unilateral vestibular hypofunction. VOR, Vestibulo-ocular reflex. PPV, Positive predictive value. NPV, Negative predictive value.

## Data Availability

The anonymized data supporting the findings of this study are available as Supplementary Material.

## Supplementary Materials

The following supporting information can be downloaded at: https://www.mdpi.com/article/10.3390/jcm15176882/s1.

## Abbreviations

The following abbreviations are used in this manuscript:

VOR	Vestibulo-ocular reflex
vHIT	Video head impulse test
UVH	Unilateral vestibular hypofunction
SPV	Slow-phase velocity
CP	Canal paresis
PPV	Positive predictive value
NPV	Negative predictive value
